# Giant hydronephrosis secondary to an ectopic ureter associated with bilateral duplex collecting system: a case report

**DOI:** 10.1093/omcr/omac034

**Published:** 2022-04-19

**Authors:** Muhamad Sinan Muhamad, Mohammad Anas Mousa, Majdy Oukan, Ali Razzok

**Affiliations:** Division of Urology, Department of Surgery, Tishreen University Hospital, Latakia, Syria; Division of Urology, Department of Surgery, Tishreen University Hospital, Latakia, Syria; Division of Urology, Department of Surgery, Tishreen University Hospital, Latakia, Syria; Division of Urology, Department of Surgery, Tishreen University Hospital, Latakia, Syria

## Abstract

Giant hydronephrosis is an ultimate rare urologic entity; even rarer when it is secondary to a duplex collecting system. Duplex collecting system is a common urologic anomaly with a wide range of clinical symptoms and a variety of associated urologic abnormalities such as an ectopic ureter, ureterocele, vesicoureteral reflux and ureteropelvic junction obstruction. This report presents a case of an 8-year-old boy who had a bilateral duplex collecting system that was revealed accidentally by a bilateral severe hydronephrosis. The duplication was complete on the left side and partial on the right with a right ectopic ureteral orifice, in addition to a bilateral vesicoureteral reflux. The vesicoureteral reflux retreated completely in the left side after using a urethral catheter for 6 months, while the decision of performing a surgical operation for the right side was made.

## INTRODUCTION

Giant hydronephrosis defines a collecting system that contains more than a litre of fluid [1]. Giant hydronephrosis has become ultimately rare to witness, this is mainly attributed with the great developments of diagnosing methods and healthcare services. Giant hydronephrosis mainly occurs as a result of an ureteropelvic junction obstruction (UPJO) and is rarely associated with a collecting system duplication [2]. Duplex collecting system is one of the most common urologic anomalies, it might be unilateral or bilateral, complete or partial and it may be associated with other abnormalities such as ureterocele, ectopic ureter, vesicoureteral reflux (VUR) or UPJO [3]. In this case, we will present a bilateral duplex collecting system; complete on the left side and partial on the right with a right ectopic ureteral orifice, in addition to bilateral VUR, which was accidently revealed by giant hydronephrosis.

## CASE REPORT

An 8-year-old boy was hospitalized due to abdominal and flank pain accompanied with hyperthermia, fatigue and general malaise. Patient history included recurrent pyelonephritis. Physical examination showed a distended non-tender abdomen; on percussion, a dull sound was seen with no shifting dullness elicited. Laboratory tests were performed, which included a full blood count, blood biochemistry studies and urinalysis (Table 1).

**Figure1:**
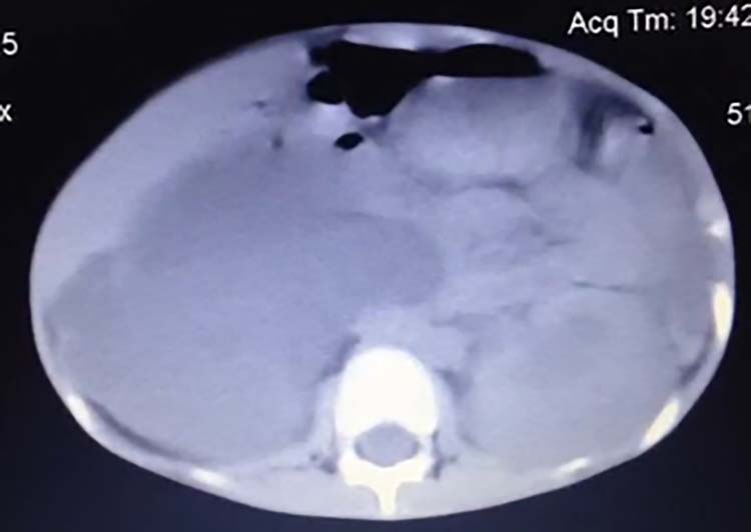
A CT scan of the abdomen and pelvis revealed giant hydronephrosis of the right kidney and mild hydronephrosis of the left kidney.

The patient underwent an abdominal ultrasound, which showed a large amount of fluid occupying the right abdominopelvic region, and a left kidney with mild hydronephrosis, the right kidney was not detected. A CT scan of the abdomen and pelvis was performed, which revealed a giant hydronephrosis in the right kidney and a moderate hydronephrosis in the left kidney (Fig. 1). Therefore, a bilateral nephrostomy was done, where almost 2 L of turbid urine were drained out from the right kidney alone.

The patient was admitted to ICU for 48 h for observation and was hospitalized for another 5 days in the ward, then he was discharged home after his condition improved significantly. A month after, our patient underwent another set of blood tests where all of them were normal (Table 1).

**Figure 2:**
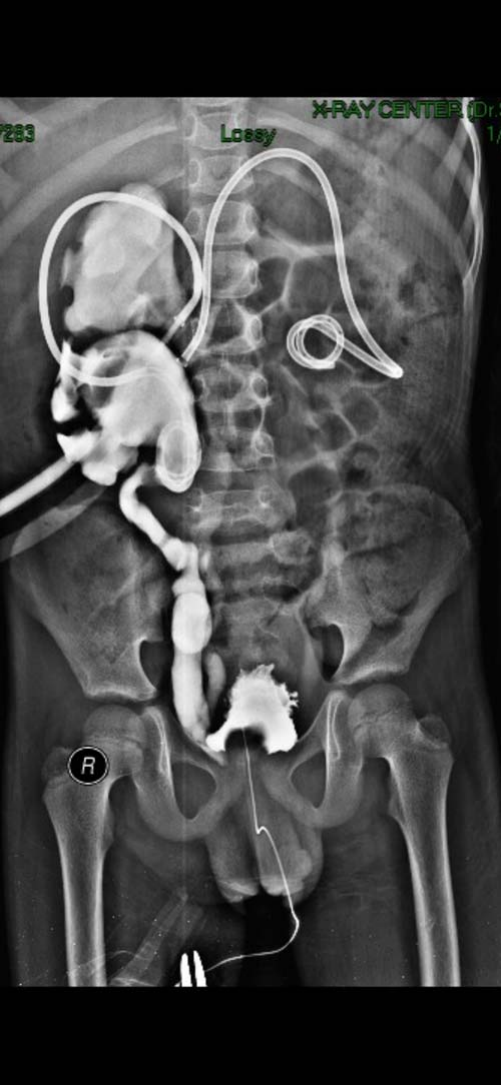
Simultaneous IVP and right side antegrade pyelography, partial duplex collecting system in the right side is detected.

Antegrade imaging (by the nephrostomy tubes) and voiding cystourethrography (VCUG) were performed, (revealing bilateral duplex collecting system; complete on the left side, and partial on the right where the two ureters fuse almost 1 cm away from the ureteral orifice). Also a bilateral VUR was detected; grade III on the left side ureter and grade V on the right side (Fig. 2).

**Table 1:** The laboratory tests when the patient was hospitalized

Laboratory tests at admission		Laboratory test after a month of treatment	
Creatinine mg/dL	8.5	Creatinine mg/dL	0.8
Urea mg/dL	193	Urea mg/dL	22
CRP mg/L	94	CRP mg/L	4.9
WBCs 109/L	22.72	WBCs 109/L	5.5
Neutrophils %	85.9%	Neutrophils %	64.1
HGB g/dL	8.2	HGB g/dL	12.1

**Figure 3:**
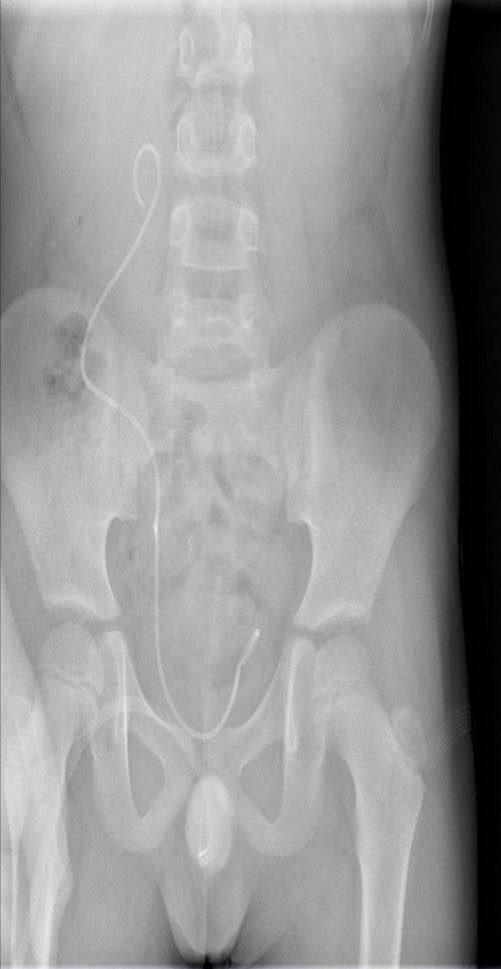
A JJ catheter was installed in the right ureter.

Cystoscopy was performed later to detect the presence of other associated abnormalities, where a right ectopic ureteral orifice located in the prostatic urethra was found. Both of the nephrostomy tubes were removed, with a JJ catheter being installed in the right ureter to prevent hydronephrosis recurrence (Fig. 3), as well as the placement of a temporary urinary catheter for 6 months to prevent the VUR occurrence in both sides.

Six months after, another VCUG was performed, the VUR retreated completely on the left side, while it remained on the right.

**Figure 4:**
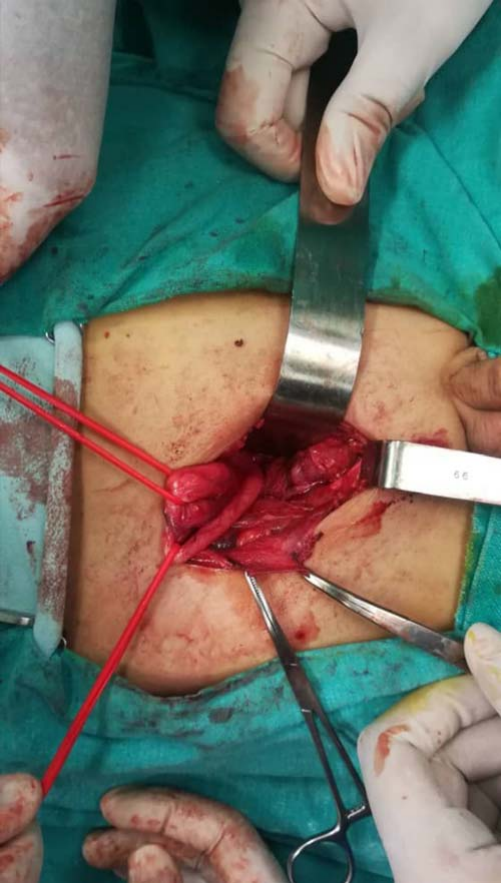
The fusion of both right ureters was almost 1 cm away from ureteral orifice.

As a result, the decision was made for a surgical operation (reimplantation of the right ureter using an anti-reflux method on the bladder and a termino-lateral anastomosis of the lower right pole ureter on it). The operation was performed (Fig. 4), and then the patient was discharged home after his general condition stabilized. Six months after, a cystoscopy and VCUG were performed showing no hydronephrosis or VUR in both sides.

## DISCUSSION

Duplex collecting system is a congenital abnormality in which a variety of complete and incomplete duplications of the collecting system happen as a result to an incomplete fusion of upper and lower pole moieties. It is one of the most common urological anomalies, although it is considered to be rare (0.8%) in comparison with other congenital deformities of the body [4]. Females are affected more than males (2:1). The duplication might be unilateral (more common) or bilateral, it may be either complete (less common) or partial [5].

Duplicated collecting system could be associated with other deformities such as ureterocele, UPJO, VUR and ectopic ureter which is the most common [6]. A various range of symptoms might manifest according to the associated distortions and the case severity. The vast majority of cases are being diagnosed in early childhood [7]. However, a large number of patients are asymptomatic and diagnosed incidentally. The most important and common symptoms are those related to VUR and urinary tract infections. In some extremely rare cases, a giant hydronephrosis is present, where a hydronephrotic kidney contains >1 L of fluid which could result in flank pain and a palpable mass.

In all cases, imaging is mandatory to confirm the diagnosis and uncover the associated deformities. Although the ultrasound provides excellent anatomic information, it struggles to detect the duplication and the related abnormalities which CT could decisively delineate. Intravenous pyelogram (IVP) could assess the renal function and how it is affected by the duplication. VCUG helps in detecting VUR specifically. Cystoscopy may be done as well to look for any accompanied anomalies such as an ectopic ureter.

The patient from our case report had a complete left side duplicated collecting system associated with VUR and moderate hydronephrosis, and no surgery was done on this side because the hydronephrosis and the VUR fully subsided after indwelling a urinary catheter; this was confirmed by the patient’s follow-up. On the right side, a partial duplication was confirmed where two ureters arise from two separate pelvises and fuse 1 cm away from the ectopic orifice, which is located in the prostatic urethra. Considering the facts of the right kidney’s unresponsive giant hydronephrosis and the persistence of the VUR after 6 months of conservative treatment, the decision to perform a surgical operation was made. The upper pole ureter was reimplanted into the bladder wall using an anti-reflux technique, whereas the lower pole ureter was anastomosed on it by performing a termino-lateral anastomosis.

## CONCLUSION

Despite the wide prevalence of the collecting systems doubling, the diversity of the accompanying deformities and the great difference in case severity and clinical manifestations make it an interesting case always, which should be carefully studied to achieve the best possible management.
